# Binocular Flash Suppression in the Primary Visual Cortex of Anesthetized and Awake Macaques

**DOI:** 10.1371/journal.pone.0107628

**Published:** 2014-09-12

**Authors:** Hamed Bahmani, Yusuke Murayama, Nikos K. Logothetis, Georgios A. Keliris

**Affiliations:** 1 Max Planck Institute for Biological Cybernetics, Tübingen, Germany; 2 Bernstein Center for Computational Neuroscience, Tübingen, Germany; 3 Imaging Science and Biomedical Engineering, University of Manchester, Manchester, United Kingdom; University College London, United Kingdom

## Abstract

Primary visual cortex (V1) was implicated as an important candidate for the site of perceptual suppression in numerous psychophysical and imaging studies. However, neurophysiological results in awake monkeys provided evidence for competition mainly between neurons in areas beyond V1. In particular, only a moderate percentage of neurons in V1 were found to modulate in parallel with perception with magnitude substantially smaller than the physical preference of these neurons. It is yet unclear whether these small modulations are rooted from local circuits in V1 or influenced by higher cognitive states. To address this question we recorded multi-unit spiking activity and local field potentials in area V1 of awake and anesthetized macaque monkeys during the paradigm of binocular flash suppression. We found that a small but significant modulation was present in both the anesthetized and awake states during the flash suppression presentation. Furthermore, the relative amplitudes of the perceptual modulations were not significantly different in the two states. We suggest that these early effects of perceptual suppression might occur locally in V1, in prior processing stages or within early visual cortical areas in the absence of top-down feedback from higher cognitive stages that are suppressed under anesthesia.

## Introduction

Visual information is processed across a distributed network of interconnected visual areas [1]. The primary visual cortex (V1), being hierarchically the first cortical area receiving information from the eyes through the thalamus, constitutes a cornerstone of the visual system [2], [3], [4], [5], [6]. Although V1 has been studied extensively and is arguably the best-understood area in the cerebral cortex, its role in visual awareness remains controversial and has been a subject of intense debate [7], [8], [9], [10], [11], [12], [13].

The use of visual stimuli that induce ambiguous perception has been established as a classical paradigm to identify the neural circuits subserving subjective perception [14], [15], [16], [17], [18]. Under these conditions, a single interpretation of the external world cannot be unambiguously achieved. When the brain is presented with such stimuli, typically only one possible interpretation is perceived at a time and after a few seconds the percept switches abruptly to another [18]. Notably, such perceptual alternations occur while the sensory input is kept constant, thus offering a clear dissociation of sensory stimulation and subjective awareness [9], [11], [14], [19], [20], [21]. Some celebrated examples of such perceptual phenomena include binocular rivalry (BR) and binocular flash suppression (BFS) [22], [23], [24], [25], [26], [27], [28]. Based on many psychophysical studies over decades, the primary visual cortex (V1) was implicated as an important candidate for the site of perceptual suppression during BR [29], [30], [31], [32], [33]. However, neurophysiological evidence obtained in monkeys did not corroborate this hypothesis but instead found only a small percentage of neurons that modulated their activity in parallel with the subjective perception of the animals [10], [19], [34], [35], [36], [37]. In contrast, studies using functional magnetic resonance imaging (fMRI) in humans found that V1 is indeed modulated to a large extent by the subjective percept [12], [13], [38], [39], [40].

Possible explanations for this discrepancy include differences in the stimulus configurations, the species tested, and the experimental methodology. In addition, a major difference between many of these studies is the extent to which the subject is involved in attending and consciously reporting the bistable alternations [41], [42], [43]. Such higher cognitive processes could be based on different mechanisms from those subserving local processes and are only observable in V1 when the subject is awake and behaving [44], [45].

In order to disentangle these two processes and investigate the role of the local processing during multistable stimulation, we performed and compared BFS experiments in anesthetized and awake, passively fixating macaques. We conjectured that any effects preserved under anesthesia, in the absence of cognitive feedback from central processes, might reflect local interactions critically involved in the initiation of competition during incongruent stimulation. We found comparable modulations in neural activity as reflected in the multi-unit activity (MUA) and local field potentials (LFPs) in both anesthetized and awake passively fixating animals. Our results suggest that the small significant modulations observed under these non-attentive conditions are arising from circuit mechanisms in early visual areas. It remains to be shown if top-down feedback to V1 engaged during active behavior would elicit larger modulations comparable to the ones previously reported by fMRI.

## Materials and Methods

### Ethics Statement

The experimental and surgical procedures were performed with great care and in full compliance with the German Law for the Protection of Animals, the European Community guidelines for the care and use of laboratory animals (EUVS 86/609/EEC), and the recommendations of the Weatherall report for the use of non-human primates in research (http://www.mrc.ac.uk/Utilities/Documentrecord/index.htm?d=MRC003440). The regional authorities (Regierungspräsidium Tübingen) approved our experimental protocol (Nr. KY1/02) and the institutional representatives for animal protection supervised all procedures. Animals were kept in large cages located adjacent to the training and experimental facilities. Space in these cages allows swinging and jumping, and enrichment equipment such as toys was changed frequently. Group housing was maintained to increase the quality of life by rich visual, olfactory, auditory and social interaction and stimulation for play. Balanced nutrition and regular veterinary care and monitoring, were provided. Chamber implantation and an anatomical scan were performed while the animals were under general anesthesia and aseptic conditions. To alleviate post-surgical pain we administered analgesics for a week after the surgery (see also surgical procedures below). Animals were not sacrificed after the experiments.

### Subjects

Four adult monkeys (*Macaca mulatta*) where used for anesthetized (N = 2; B01 and D01, 6 years old, weighing 10 and 8 kg respectively) and awake (N = 2; D98 and F03, aged 12 and 9 years, weighing 16 and 11 kg respectively) electrophysiological recordings.

### Surgical procedures

Recording chambers were positioned over the operculum in area V1 according to stereotaxic coordinates. This was aided by high-resolution magnetic resonance anatomical imaging. The anatomical scan and recording chamber implantation were done while the animals where under general anesthesia. Details of the procedure can be found elsewhere [37], [46].

### Visual stimulation and data acquisition

#### Anesthetized experiments

Data was recorded from two monkeys (B01 and D01) in separate sessions (two sessions for monkey B01, three sessions for monkey D01) under general anesthesia. The procedure is described in detail previously [46]. Balanced anesthesia was maintained with isoflurane (end-tidal 0.3%) and fentanyl (3 µg/kg/hr). Muscle relaxation was achieved with mivacurium chloride (3–6 mg/kg/hr). Physiological parameters were monitored and maintained within the normal physiological range [46].

Visual stimuli were presented binocularly using a SVGA fiber optic system (Avotec, Silent Vision) with a resolution of 800×600 pixels at 60 Hz frame rate. To focus the eyes on the stimulus plane, animals were fitted with eye-lenses (Woehlk-Contact-Linsen). The eyepieces of the presentation system were positioned by using a modified fundus camera (Zeiss RC250) which ensures the alignment of the stimulus center with the fovea of each eye [46]. The size of stimuli varied between 6° to 9° radius in different sessions.

Intracortical recordings were conducted with the Eckhorn multielectrode arrays [47], [48]. This allowed us to simultaneously monitor and record from up to 13 sites. Electrodes were Pt_90_W_10_ wire (20 µm diameter) with a glass coating (80 µm external diameter) and were guided into the brain through the overlying dura mater. During the recordings, a custom-made adaptor was used to distribute the electrodes against the dura in a 4×4 square array, with an inter-electrode spacing of 2.5 mm which separated the neighboring electrode pairs by 2.5 mm, while the pairs on opposite corners had a physical separation of 10.6 mm.

Data collection was controlled by an industrial PC (Advantech) running under the QNX operating system (QNX Software Systems). The broadband signals from each channel were amplified by a factor of 8000 and band-pass filtered between 1 Hz and 5 kHz (Alpha Omega Engineering). The signals were then individually digitized at a rate of 20.83 kHz on a 16-bit analog to digital board (PCI-6052E; National Instruments) and stored on a PC for further analysis using custom software written in MATLAB (The Mathworks Inc.).

#### Awake experiments

Two other animals (D98 and F03) were used in the awake sessions. We recorded spiking activity as well as local field potentials (LFP) from V1 of both monkeys by custom made tetrodes guided to the brain by manually adjustable microdrives (Crist Instrument Co.). We also recorded from a chronically implanted array of tetrodes inside a form-specific titanium chamber over the operculum of one of the monkeys (D98). The recording chambers were from either medical-grade titanium or polyether ether ketone (TECAPEEK; Ensinger GmbH). Details have been described elsewhere [37], [49]. The animals were implanted with a scleral search coil [50], [51] and their eye movements were monitored online.

Visual stimuli were sinusoidal gratings with different orientations and a typical size of 1°–2° in diameter, displayed using a dedicated graphics workstation (TDZ 2000; Intergraph Systems) running an OpenGL-based stimulation program. Dichoptic presentation of the visual stimuli was through a custom-made stereoscope with two LCD monitors at both sides running at a resolution of 1280×1024 and a 60 Hz refresh rate. After eye calibration and alignment of the displays, a coarse receptive field mapping was performed to position the stimulus for the experiments. The multi-unit responses were put through a sound amplifier (Grass Technologies) so that the experimenter could evaluate the gross location of the receptive fields and the preferences of the multi-unit responses towards different orientations and sizes. Details have been described previously [37].

Multi-unit activity was sampled at 32 kHz, digitized (12 bits), and stored using the Cheetah data acquisition system (Neuralynx) and was defined as the events that exceeded a predefined threshold (25 µV) of the filtered (600 Hz–6 kHz) and digitized signal. LFP signals were recorded after filtering the raw signal using analog band-pass filtering (1 Hz–475 Hz) and digitized at 2 kHz (12 bits).

### Experimental design

To study the relationship between neural activity and perceptual modulations, we used the paradigm of binocular flash suppression (BFS). During this paradigm, a visual stimulus presented to one eye is suppressed from awareness as a result of presenting a different stimulus, referred to as *flash*, to the other eye at the location corresponding to the image to the first eye [26].

**Figure 1:**
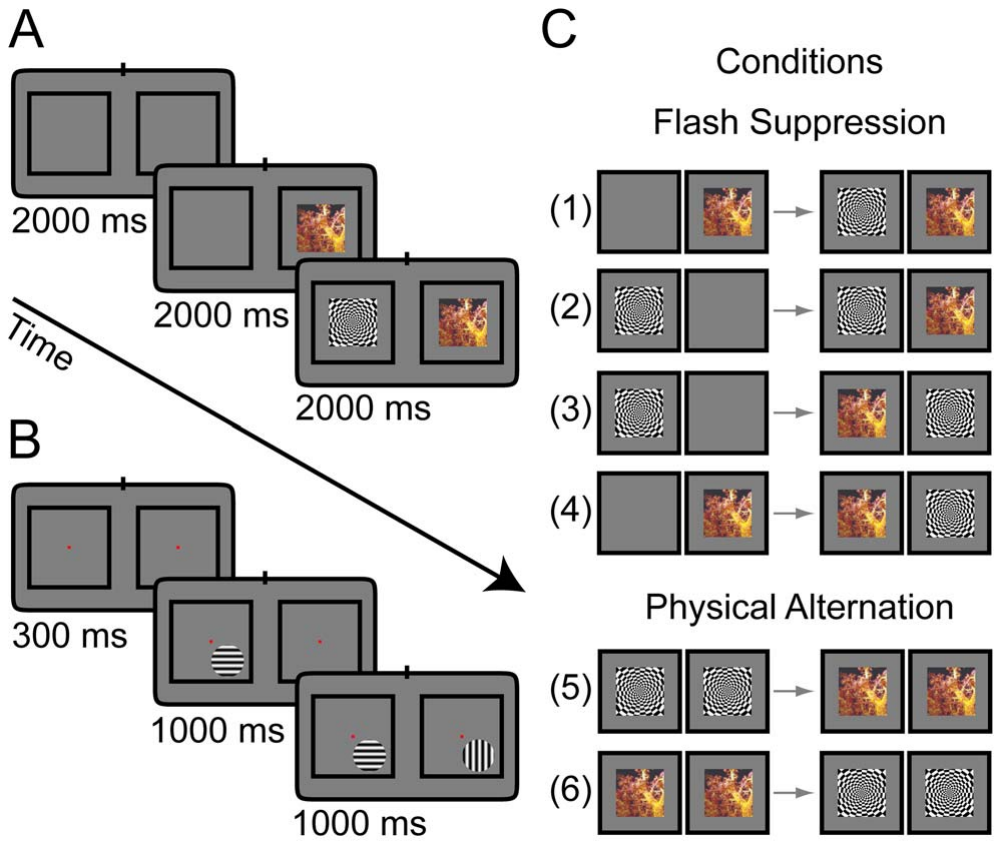
Illustration of the binocular flash suppression (BFS) paradigm for the anesthetized and awake experiments. (**A**) The sequence of presentation of the two stimuli to the two eyes in the anesthetized experiments. An intertrial interval of 2000 ms was followed by a stimulus presented in corresponding locations to one of the two eyes for 2000 ms. A second stimulus was added to the other eye for another 2000 ms. Stimuli have been chosen from a battery of natural and synthetic images to elicit maximal difference in the neural activity. (**B**) Sequence of BFS presentation in the awake experiments. After an intertrial period (1000–3000 ms) the animal fixated on a central point for 300 ms in order to initiate the trial and then monocular and binocular stimuli followed similar to **A**. Stimulus presentation times were 1000 ms and the stimuli were static sinusoidal gratings with orthogonal orientations optimized to elicit maximal (preferred orientation) and minimal (non-preferred orientation) responses in the recorded channels. The position of the stimuli was chosen to cover the receptive fields of the recorded sites and the sizes of the stimuli were 1°–3°. The animal fixated within a window with radius 0.5° around a small point throughout the trial to receive juice reward. Unsuccessful trials were aborted and not further analyzed. (**C**) Different possible configurations of BFS and control conditions. Left and right columns present the monocular and binocular periods respectively. Note that the four different flash suppression conditions can be split in two pairs (1–2 and 3–4) with identical stimulus presentation across the different eyes albeit different perceptual outcomes depending on the initial monocular stimulus (see methods). Physical alternation conditions (5–6) demonstrate an identical perceptual experience albeit without binocular conflict and serve as controls.

In anesthetized experiments, monkeys were presented with blank screen for two seconds in the beginning of each trial. Subsequently, one of the two stimuli was presented alone to either the left or the right eye for two seconds, followed by the onset (flash) of the second stimulus at the corresponding retinal location in the contralateral eye and the simultaneous presentation of both incongruent stimuli for another two seconds until the end of the trial. The two stimuli have been chosen to elicit maximal differences in neural activity based on the average responses to a battery of natural and generic images (N = 50) that were presented to the animal prior to the BFS experiment. The time course of an example trial is depicted in Figure 1A for two stimuli used in our experiments. Figure 1C shows all four possible configurations of BFS conditions (1–4) as well as two control conditions termed physical alternation (5–6). For these conditions, the stimuli are presented congruently across the two eyes producing the same perceptual sequence (in awake conditions) and are used as controls.

The same paradigm was used in awake experiments. The monkeys had to passively fixate on a central fixation point in order to initiate the BFS trial. A fixation point (0.2°) appeared in the center of the screen for 300 milliseconds followed by flash suppression stimulation similar to anesthetized condition but with a duration of one second for each period (Fig. 1B). Stimuli were static sinusoidal gratings with orthogonal orientations optimized to elicit maximal differences between the responses to the two orientations. The sizes of the stimuli were 1°–3°, covering the receptive fields of the recorded sites. A drop of juice was delivered to the animal if it maintained the fixation throughout the trial. For more details see [37].

### Statistical and data analysis

We used custom programs written in Matlab (The Mathworks Inc.) for data analysis. Statistical significance (P<0.05) of physical and perceptual modulations was assessed by using a nonparametric Wilcoxon rank sum test for equal medians. For all the comparisons, we excluded the first 500 ms after the flash to avoid initial transient biases. We then calculated the preference and modulation indices by using discriminability index (*d′*). This was defined as:
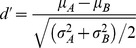
Where *μ* and *σ* are the mean response and the standard deviation of the conditions put in the comparison. In this paper, we report the *d′* indices for either pairs of binocularly presented identical stimuli (referred to as sensory preference *d′_sens_*) or incongruent stimuli (referred to as perceptual preference *d′_perc_*). Bigger values of *d′* indices indicate larger discriminability of responses and thus larger preference to one of the conditions being compared.

To estimate the time-courses of neural adaptation to prolonged presentation of the preferred stimulus we fitted exponentials of the form 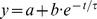 to the data of the monocular period.

## Results

### Perceptual modulation of multi-unit activity

We recorded neural activity from V1 of four macaques being either under general anesthesia (B01 and D01), or awake, passively fixating (D98 and F03). This allows the comparison of neural activity in the anesthetized and awake brains during the BFS task, which in awake conditions, ensures robust perceptual suppression of a monocular stimulus upon asynchronous presentation of a second stimulus to the other eye (see Materials and Methods for details). Recordings were performed with the Eckhorn multi-electrode arrays and custom made tetrodes. Unless otherwise specified, statistical tests were performed using a Wilcoxon two-sided rank sum test with a critical value of 0.05.

**Figure 2:**
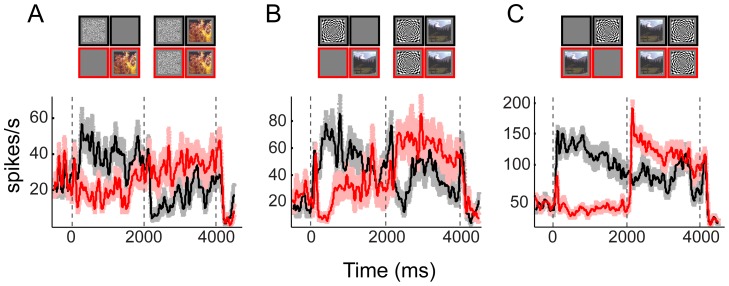
Examples of multi-unit modulations during binocular flash suppression in anesthetized monkeys (A–C). The spike-density functions of three example multi-units are shown (A from monkey B01, B–C from monkey D01). Upper diagrams on each panel demonstrate the conditions with corresponding color outlines as the spike density functions below. The conditions contrasted in each case had the same stimulus during the binocular period but notably predict different percepts in awake subjects. Note the significant modulations during the binocular (same stimulus) conditions that are, however, smaller than the preference during the monocular presentation. The shaded areas represent SEM across trials. Time zero was defined to be the onset of the monocular stimuli.

During anesthetized experiments, we recorded multi-unit activity from 33 electrode penetrations in two monkeys. In monkey B01, 13 electrodes were used in a single experimental session while in monkey D01, 10 electrodes were used in two separate sessions. From the total of 33 MUAs in two monkeys, 31 (94%) were visually responsive. Out of these, 29 showed significant tuning to the physical alternation conditions as measured by the *d*′ index (see Materials and Methods). Twenty multi-unit sites (two non-tuned during physical alternation) showed significant modulations across at least one pair of binocular incongruent conditions with the same stimulus configuration. Note that these conditions elicit different percepts in awake subjects and we will refer to them as “perceptual” modulations but keep in mind that the animal was anesthetized. Some examples of significantly modulating sites are presented in Figure 2. On average, the magnitude of the perceptual modulations was substantially smaller 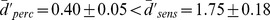 [*μ*+SEM] in comparison to the physical alternation period (*p* = 1.8×10^−8^, one-tailed two-sample T-test, N = 31 visually responsive sites).

For awake experiments, we analyzed the spiking activity recorded from two other animals (D98 and F03). Analysis of the single unit activity of these two animals has been published elsewhere [37]; here we report multi-unit activity (MUA) and compare it with MUA from the anesthetized experiments. From a total of 393 multi-units, 364 were visually responsive (92%) and 275 units showed sensory tuning to the visual stimuli. Perceptual modulations were found in 88 of these sites. As in the anesthetized, the magnitude of the perceptual modulations was substantially smaller 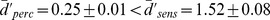 [*μ*+SEM] in comparison to the physical alternation period (*p*≈0, one-tailed two-sample T-test, N = 364 visually responsive sites).

### Comparison between the two conscious states

To study the effect of unconsciousness (during anesthesia) on perceptual modulations during flash suppression, we compared the spiking activity in anesthetized monkeys with those of their awake counterparts. Differences between the two conditions could potentially be also attributed to differences in experimental design and indirect influences of anesthesia (but see Discussion).

**Figure 3:**
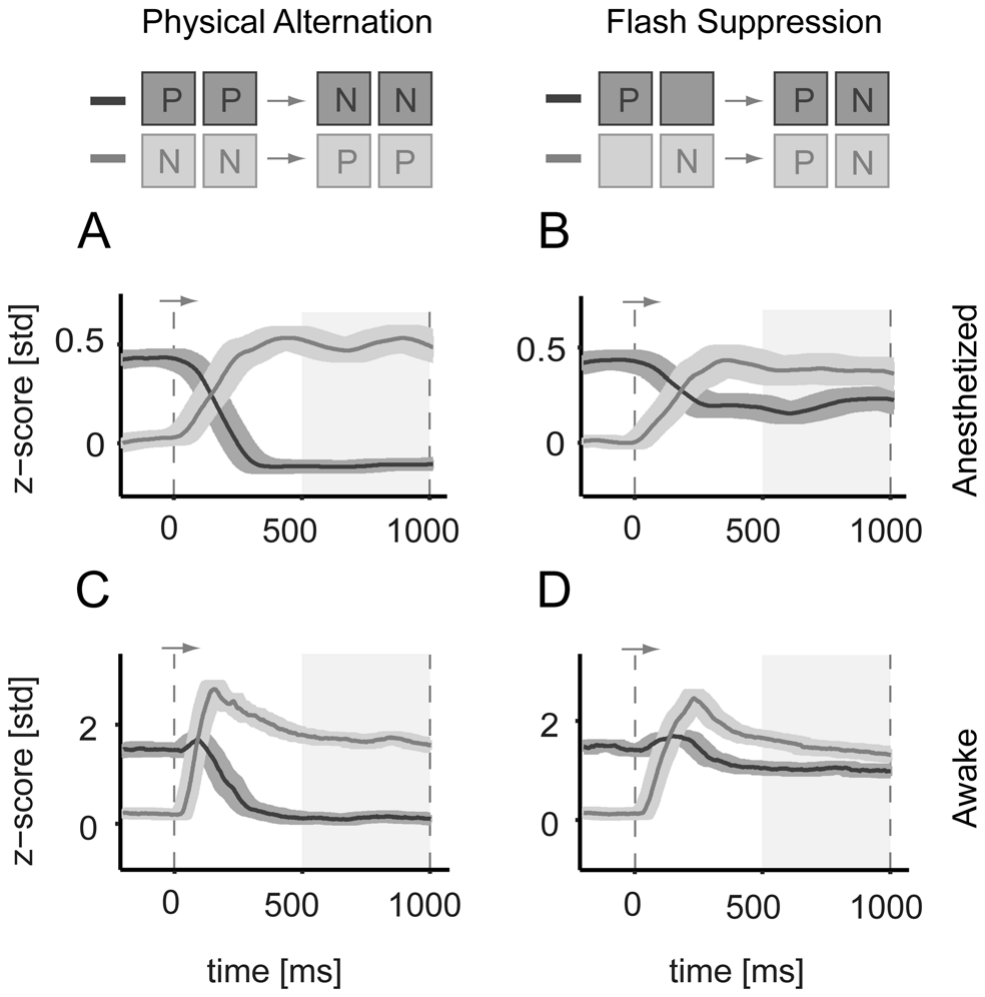
Population responses of neurons during physical alternation and binocular flash suppression conditions in anesthetized (A,B) and awake (C,D) monkeys. Different conditions are depicted on top of the panels with corresponding shaded outlines as the spike density functions below. Eyes are presented with preferred (P) and non-preferred (N) stimuli. Left group on the top are the two conditions of physical alternation and rightward the two flash suppression conditions eliciting the same perceptual sequence. (**A**) Population activity in z-scores during physical alternation conditions and (**B**) during binocular flash suppression in anesthetized monkey for all visually responsive multi-units (N = 31) in anesthetized experiments. Prior to averaging, conditions were sorted to preferred (P) and non-preferred (N) according to the responses during the monocular presentation. (**C,D**) the same as **A** and **B**, but in awake passively fixating monkeys. In all four panels, dark lines represent the responses to the preferred stimulus followed by non-preferred stimulus after the flash. Lighter gray lines represent the responses to the non-preferred stimulus first followed by the responses to the preferred in the second period. The shaded areas around the lines represent SEM across sites. Time zero was defined to be the time of the flash or switch of the stimuli.

Figure 3 compares the activity for the population of multi-units across the two conscious states. A small but significant modulation was present in both the anesthetized and awake states during the flash suppression period (Fig. 3B, D). Furthermore, the relative amplitudes of the perceptual modulations as measured by the ratio of perceptual to sensory |*d′*| were not significantly different for the two states. This was on average 28% and 25% of the sensory modulation in anesthetized and awake conditions respectively (across significantly modulating sites in both animals in each conscious state, N = 84 in awake and N = 18 in anesthetized). The close similarity between the relative amplitude of modulations in the two conditions suggests similar mechanisms for these two states.

Table 1 summarizes the numbers and percentages of significant modulations for both conscious states. We tested if the proportion of perceptually modulating sites (PM) was significantly different in anesthetized and awake macaques (two-sampled t-test between proportions, t-value 4.924, *p*<10^−5^). This was on average 65% in anesthetized (20/31 recorded units with a 95% C.I. of 48–81%, Bernoulli distribution), which was higher than the average of 24% in awake macaques (88/364 recorded units with 95% C.I. of 20–29%, Bernoulli distribution).

**Table 1:** Numbers and percentages of significant modulations.

		Anesthetized	Awake
T	Total # of multi-units/recorded sites	33	393
VR	Visually responsive (% of T)	31 (94%)	364 (92%)
SM	Sensory stimulus modulation (% VR)	29 (94%)	275 (76%)
PM	Perceptual stimulus modulation (% VR)	20 (65%)	88 (24%)
PaS	Perceptual & sensory (% PM)	18 (90%)	84 (95%)
xP	Only perceptual (% PM)	2 (10%)	4 (5%)

The absolute numbers and respective percentages of significant modulations are presented for multi-unit activities (MUA) in the two conscious states. In the first row (T) the total numbers of multi-units/recorded-sites are reported. The second row (VR) presents the number (percentage) of sites that showed significant visual responses. The third row (SM) presents the number of sites that were responding differentially two the different congruent stimuli (sensory modulation) and the fourth row (PM) the number of sites that showed differential responses under the different perceptual conditions (under the same stimulus) as a percentage of visually responsive units/sites. Note the significant difference in PM between the two conditions. In the last two rows, PaS presents the numbers of perceptually modulating sites that showed, in addition, sensory modulations and xP presents the numbers of sites that showed exclusively perceptual modulations.

### Flash suppression and adaptation

**Figure 4:**
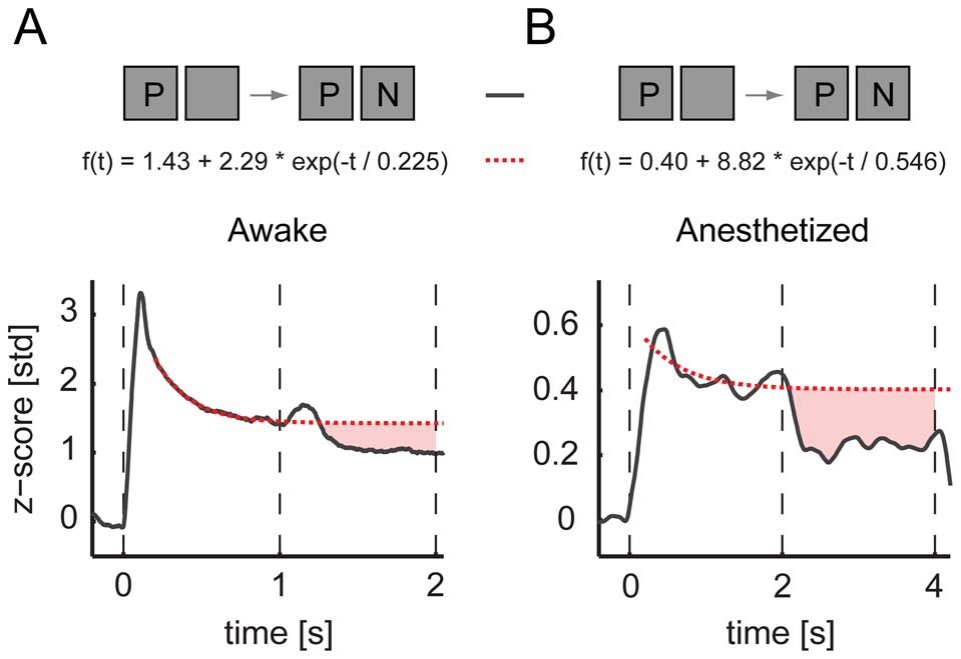
Effects of adaptation. (**A**) The suppression caused by the presence and perception of the non-preferred stimulus (N) compared with a modeled (red-dashed line) continuous presentation of the preferred stimulus (P) in awake experiments and (**B**) in anesthetized experiments. Shaded red areas indicate the additional suppression caused by interocular and/or stimulus interactions beyond adaptation.

Neural adaptation is an inherent potential complication of the binocular flash suppression (BFS). The different history of stimulation leading to the two alternative percepts can introduce differences in the level of adaptation in the neural populations encoding the competing stimuli. However, a simple model of adaptation cannot explain the responses of V1 neurons during BFS. To examine this, we estimated the time course of adaptation of the first stimulus (preferred) for the whole duration of a trial by fitting an exponential to the data during the monocular presentation (see Materials and Methods). We found that the parallel presentation of the non-preferred stimulus during BFS introduces additional suppression compared to the estimated level of activity predicted by adaptation (Fig. 4). The time constants of adaptation (τ) were τ_aw_ = 225 ms for the awake while for the anesthetized adaptation was slower with τ_an_ = 546 ms. These results demonstrate that interocular and/or stimulus interactions beyond adaptation are taking place and contribute to the perceptual modulations during incongruent stimulation. Similar interactions were also present at the level of single neurons reported in a previous study (see figure 10 in [37]).

### Modulations of the local field potentials

**Figure 5:**
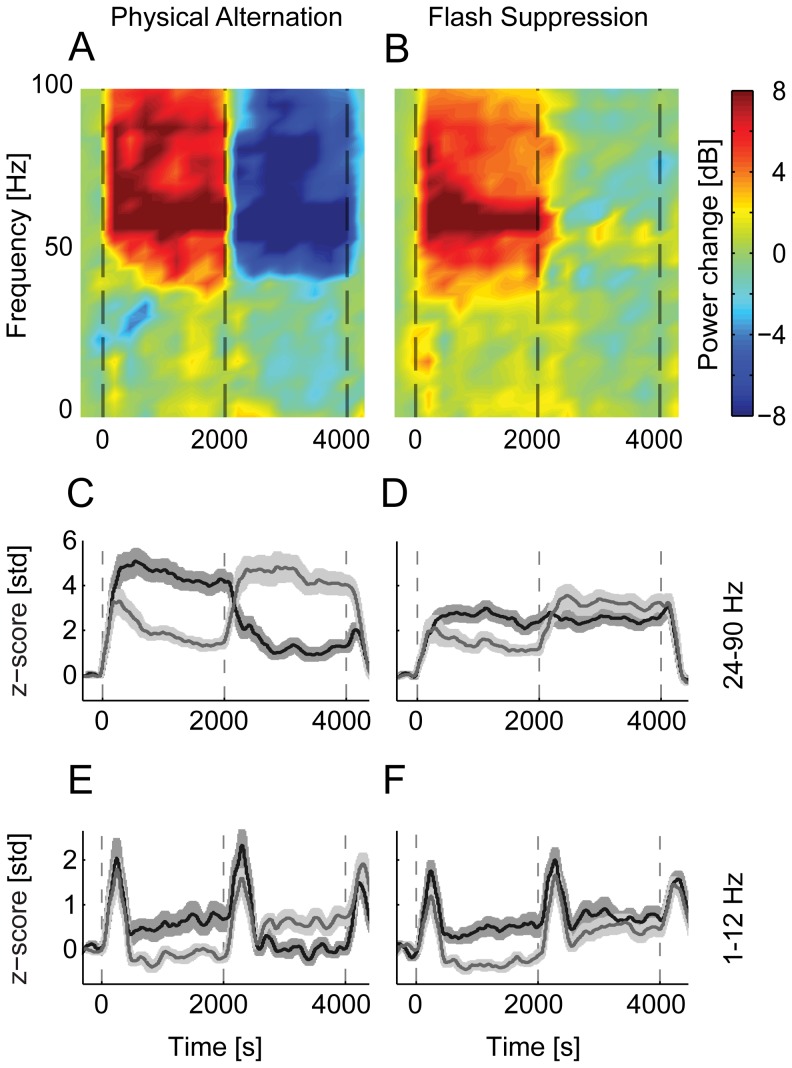
Population average of the local field potentials (LFP) of all visually responsive sites (anesthetized experiments). (**A**) The average difference in the spectrogram between the two conditions during physical alternation periods and (**B**) during flash suppression conditions. Spectrograms are plotted only for frequencies below 100 Hz. (**C,D**), Time domain band-passed average of the gamma-band frequencies (24–90 Hz) for physical alternation and flash suppression conditions. (**E,F**), Time domain average of the lower frequency bands (1–12 Hz), during physical and perceptual alternations, respectively.

We acquired local field potentials from 33 recording sites in three anesthetized experiments. Similar to the awake results reported previously [37], the power of the gamma frequency range of the LFP (24–90 Hz) showed an increase shortly after the stimulus onset. Also, a preference for the stimulus was observed in 26 recording sites during congruent stimulation. During the incongruent presentation, however, only one third of recording sites (11/33) showed a significant difference in perceptual modulation. Similar to the MUAs, this difference was substantially smaller than sensory tuning of the LFPs (Fig. 5).

Lower frequency LFP power (1–12 Hz) showed a significant increase in oscillatory activity after stimulus onset in only 14 of 33 recording sites. Sensory tuning to the stimulus was observed in the same fraction of recording sites (14). During the dichoptic phase (perceptual suppression), this difference was significant in only 4 recording sites of one of the animals (B03). These results indicate that perceptual modulations of the lower band of the LFP in V1 are essentially absent in anesthetized conditions, similar to the awake passively fixating animals reported previously [37].

## Discussion

The neural correlates of visual awareness have been attracting scientists' interest for decades. In particular, the role of primary visual cortex (V1) in perceptual rivalry has been a subject of intense debate [7], [8], [52]. On one hand, psychophysical data and the hierarchical position of V1 in the visual system initially suggested that perceptual suppression is resolved at the level of V1 through interocular competition between the two monocular channels [29], [32], [33]. Electrophysiological recordings from multiple visual areas in the brain including V1, on the other hand, provided evidence for competition happening at higher visual areas presumably between internal representations of stimuli rather than information from monocular channels in V1 [10], [14], [19], [36]. In a comprehensive review, Blake and Logothetis discussed supporting evidence for each of these alternatives and proposed a hybrid model of rivalry which involves both mechanisms of local and global processing at different hierarchical levels [11]. This model gained further support by a number of psychophysical and computational studies [53], [54], [55], [56].

Local and global processing in V1 could be based on different mechanisms. Feedback signals from extrastriate visual areas modulate V1 activity extensively. The density of such feedback projections is as much as or larger than the feedforward afferents [57]. For example, top-down attention is a process that could be mediated via such projections and has been shown to modulate V1 activity [58], [59], [60], [61], [62]. In a series of fMRI studies in human, perceptual suppression has also been found to strongly modulate BOLD activity in primary visual cortex [12], [13]. In addition, we have shown previously that the activity of some single cells in V1 also shows significant modulations during perceptual suppression induced by BFS [37]. However, only a small proportion of neurons in V1 showed these effects and importantly the amplitude of perceptual modulations was very small in comparison to the sensory preference. One possible explanation for the difference between the strength of modulations in area V1 of human and monkeys is the extent to which the subjects were asked to consciously attend to the stimuli [41], [63]. It is conceivable that the effects recorded by fMRI in humans reflect top-down modulations mediated by changes in attentional state and/or the active employment of the subject in the task instead of directly reflecting the competition happening at the level of V1 circuitry [39], [41], [64], [65]. On the contrary, most monkey electrophysiology studies used paradigms with animals passively fixating not directly being engaged in reporting the perceptual transitions.

Two studies that did require the monkeys to report their subjective perception also reported small percentages of neurons showing spike rate modulations [10], [35]. Note, however, that these studies used different stimulus paradigms and could potentially underestimate the effects. The first study by Leopold and Logothetis, 1996 used binocular rivalry (BR). Although BR has many advantages over BFS, the variability in the animal reaction times is expected to smooth the average triggered responses thereby underestimating the effect. The second study by Wilke et al., 2006 used generalized flash suppression (GFS) that notably involves no direct interocular interaction of corresponding retinal locations. Critically, the effectiveness of GFS depends on parameters like the distance of the surround stimuli to the target, the density etc. (see [66]). The authors adjusted the parameters so that the target would disappear only in about 50% of the trials. This means that the suppression-inducing stimulus was not as potent as in the case of BFS, for which suppression happens essentially in 100% of the trials [37], and therefore could also underestimate the effect.

In the present study, we compared neural activity in V1 during binocular flash suppression in anesthetized and awake monkeys to shed light on the mechanisms of perceptual suppression. Some aspects of global processing such as the attentive and conscious analysis of a scene have been observed in V1 only in awake and perceiving animals [64], [67], [68]. We conjectured that if the small effects observed in previous electrophysiological studies are due to influences from central processes these modulations should be eliminated under anesthesia. However, we found significant modulations of the multi-unit activity recorded in V1 of anesthetized macaques during binocular flash suppression. These modulations were small, albeit comparable to those observed in passively fixating awake animals. This suggests that these effects are arising from early processes that initiate the competition between monocular channels and do not necessarily need consciousness.

The effects of anesthesia on consciousness are controversial. Anesthesia disrupts cortical integration [69] which is associated with unconsciousness. In particular, it abolishes contextual and attentional modulation of firing, presumably mediated by feedback connections [70]. In this study, we employed an optimized protocol for balanced anesthesia that allows robust and reproducible activation of primary visual cortex and a number of extrastriate visual areas, including areas in the superior temporal sulcus [46]; however, cognitive signals like top-down attention and post-perceptual feedback to early visual areas were presumably suppressed in this state. Given the similarly small magnitudes of perceptual modulation during the awake, passively fixating condition and the anesthetized condition, we suggest that such cognitive signals from task-related central processes are a key ingredient of the larger modulations that have been observed in human V1 by fMRI.

Furthermore, we found that the proportion of perceptually modulating sites during wakefulness was significantly lower than that under anesthesia. We note that this result should be interpreted with caution as the difference could be attributed to several potential confounds such as stimulus differences and biases in electrode positioning. Another possible explanation is that processing during awake stimulation conditions might be actively reducing perceptual modulations e.g. by decorrelating the neuronal ensembles involved in the competition. For example, neuromodulatory processes like attention have been shown to decrease correlated variability in neuronal populations [71], [72], [73]. Such active gain mechanisms can largely change spiking correlations [74]. Such processes can enrich the information carried by neuronal populations in V1 and at the same time reduce bottom-up competition as reflected in perceptual modulations in multi-unit sites. Alternatively, this difference could be arising from factors that are not inherently related to perception.

In a recent study, Wilke and colleagues demonstrated that the low frequencies of the LFP in the thalamus show robust perceptual modulations only if the animals are actively reporting their percept and are eliminated when the animals passively fixate [75]. Similarly, our previous study in passively fixating animals found negligible perceptual modulations of the LFP at this frequency range [37]. Here, we found that lower frequency LFPs in anesthetized V1 were also not predictive of the percept consistent with the hypothesis that active engagement of the animal in the task might be necessary. Given the relationship between the BOLD signal and the LFP [76], it is conceivable that strong BOLD activation in V1 during binocular rivalry in humans is more likely related to the feedback from higher cognitive central stages. However, differences in the nature of the read-out signals could be still a possible explanation for these differences.

Our results confirm that V1 is involved in the process of perceptual suppression during interocular incongruent stimulation and we suggest that it plays a role in initiating the competition. This process is independent of the feedback from higher areas when the subject is not consciously involved in the task. It remains to be shown if a more pronounced and robust modulation of V1 is present during an active task, which can be eliminated under anesthesia or a no-task, passive fixation condition.

## References

[1] Felleman DJ, Van Essen DC (1991) Distributed hierarchical processing in the primate cerebral cortex. Cereb Cortex 1: 1–47. 1822724 10.1093/cercor/1.1.1-a

[2] Hubel DH, Wiesel TN (1962) Receptive fields, binocular interaction and functional architecture in the cat's visual cortex. J Physiol 160: 106–154. 14449617 10.1113/jphysiol.1962.sp006837 PMC1359523

[3] Hubel DH, Wiesel TN (1977) Ferrier lecture. Functional architecture of macaque monkey visual cortex. Proc R Soc Lond B Biol Sci 198: 1–59. 20635 10.1098/rspb.1977.0085

[4] Gilbert CD (1993) Circuitry, architecture, and functional dynamics of visual cortex. Cereb Cortex 3: 373–386. 8260807 10.1093/cercor/3.5.373

[5] Angelucci A, Bressloff PC (2006) Contribution of feedforward, lateral and feedback connections to the classical receptive field center and extra-classical receptive field surround of primate V1 neurons. Prog Brain Res 154: 93–120. 17010705 10.1016/S0079-6123(06)54005-1

[6] Sincich LC, Horton JC (2005) The circuitry of V1 and V2: integration of color, form, and motion. Annu Rev Neurosci 28: 303–326. 16022598 10.1146/annurev.neuro.28.061604.135731

[7] Crick F, Koch C (1995) Are we aware of neural activity in primary visual cortex? Nature 375: 121–123. 7753166 10.1038/375121a0

[8] Pollen DA (1995) Cortical areas in visual awareness. Nature 377: 293–295. 7566083 10.1038/377293b0

[9] Logothetis NK (1998) Single units and conscious vision. Philos Trans R Soc Lond B Biol Sci 353: 1801–1818. 9854253 10.1098/rstb.1998.0333 PMC1692419

[10] Leopold DA, Logothetis NK (1996) Activity changes in early visual cortex reflect monkeys' percepts during binocular rivalry. Nature 379: 549–553. 8596635 10.1038/379549a0

[11] Blake R, Logothetis NK (2002) Visual competition. Nat Rev Neurosci 3: 13–21. 11823801 10.1038/nrn701

[12] Tong F, Engel SA (2001) Interocular rivalry revealed in the human cortical blind-spot representation. Nature 411: 195–199. 11346796 10.1038/35075583

[13] Polonsky A, Blake R, Braun J, Heeger DJ (2000) Neuronal activity in human primary visual cortex correlates with perception during binocular rivalry. Nat Neurosci 3: 1153–1159. 11036274 10.1038/80676

[14] Leopold DA, Logothetis NK (1999) Multistable phenomena: changing views in perception. Trends Cogn Sci 3: 254–264. 10377540 10.1016/s1364-6613(99)01332-7

[15] Logothetis NK (1999) Vision: a window on consciousness. Sci Am 281: 69–75. 10920769

[16] Rock I (1995) Perception: Scientifc American Library.

[17] Rock I, Hall S, Davis J (1994) Why do ambiguous figures reverse? Acta Psychol (Amst) 87: 33–59. 7985524 10.1016/0001-6918(94)90065-5

[18] Attneave F (1971) Multistability in perception. Sci Am 225: 63–71. 5116412

[19] Logothetis NK, Schall JD (1989) Neuronal correlates of subjective visual perception. Science 245: 761–763. 2772635 10.1126/science.2772635

[20] Pitts MA, Nerger JL, Davis TJ (2007) Electrophysiological correlates of perceptual reversals for three different types of multistable images. J Vis 7: 6. 10.1167/7.1.6 17461674

[21] Ramachandran VS, Anstis SM (1985) Perceptual organization in multistable apparent motion. Perception 14: 135–143. 4069943 10.1068/p140135

[22] DuTour M (1760) Discussion d'une question d'optique [Discussion on a question of optics]. Memoires de Mathematique et de Physique Presentes par Divers Savantes. Paris: Academie des Sciences.

[23] Wheatstone C (1838) On some remarkable, and hitherto unobserved, phenomena of binocular vision. Philos Trans R Soc Lond B Biol Sci 128: 371–394.

[24] Breese BB (1899) On inhibition. Psychol Rev 3: 1–65.

[25] Breese BB (1909) Binocular Rivalry. Psychol Rev 16: 410–415.

[26] Wolfe JM (1984) Reversing ocular dominance and suppression in a single flash. Vision Res 24: 471–478. 6740966 10.1016/0042-6989(84)90044-0

[27] Tsuchiya N, Koch C (2005) Continuous flash suppression reduces negative afterimages. Nat Neurosci 8: 1096–1101. 15995700 10.1038/nn1500

[28] Tsuchiya N, Koch C, Gilroy LA, Blake R (2006) Depth of interocular suppression associated with continuous flash suppression, flash suppression, and binocular rivalry. J Vis 6: 1068–1078. 17132078 10.1167/6.10.6

[29] Blake R (1989) A neural theory of binocular rivalry. Psychol Rev 96: 145–167. 2648445 10.1037/0033-295x.96.1.145

[30] Abadi RV (1976) Induction masking–a study of some inhibitory interactions during dichoptic viewing. Vision Res 16: 269–275. 1266071 10.1016/0042-6989(76)90110-3

[31] Cogan AI (1987) Human binocular interaction: towards a neural model. Vision Res 27: 2125–2139. 3447362 10.1016/0042-6989(87)90127-1

[32] Blake R, Tadin D, Sobel KV, Raissian TA, Chong SC (2006) Strength of early visual adaptation depends on visual awareness. Proc Natl Acad Sci U S A 103: 4783–4788. 16537384 10.1073/pnas.0509634103 PMC1400587

[33] Lehky SR (1988) An astable multivibrator model of binocular rivalry. Perception 17: 215–228. 3067209 10.1068/p170215

[34] Gail A, Brinksmeyer HJ, Eckhorn R (2004) Perception-related modulations of local field potential power and coherence in primary visual cortex of awake monkey during binocular rivalry. Cereb Cortex 14: 300–313. 14754869 10.1093/cercor/bhg129

[35] Wilke M, Logothetis NK, Leopold DA (2006) Local field potential reflects perceptual suppression in monkey visual cortex. Proc Natl Acad Sci U S A 103: 17507–17512. 17088545 10.1073/pnas.0604673103 PMC1859959

[36] Sheinberg DL, Logothetis NK (1997) The role of temporal cortical areas in perceptual organization. Proc Natl Acad Sci U S A 94: 3408–3413. 9096407 10.1073/pnas.94.7.3408 PMC20383

[37] Keliris GA, Logothetis NK, Tolias AS (2010) The role of the primary visual cortex in perceptual suppression of salient visual stimuli. J Neurosci 30: 12353–12365. 20844131 10.1523/JNEUROSCI.0677-10.2010 PMC2962415

[38] Yuval-Greenberg S, Heeger DJ (2013) Continuous flash suppression modulates cortical activity in early visual cortex. J Neurosci 33: 9635–9643. 23739960 10.1523/JNEUROSCI.4612-12.2013 PMC3760788

[39] Maier A, Wilke M, Aura C, Zhu C, Ye FQ, et al (2008) Divergence of fMRI and neural signals in V1 during perceptual suppression in the awake monkey. Nat Neurosci 11: 1193–1200. 18711393 10.1038/nn.2173 PMC2754054

[40] Lee SH, Blake R (2002) V1 activity is reduced during binocular rivalry. J Vis 2: 618–626. 12678633 10.1167/2.9.4

[41] Watanabe M, Cheng K, Murayama Y, Ueno K, Asamizuya T, et al (2011) Attention but not awareness modulates the BOLD signal in the human V1 during binocular suppression. Science 334: 829–831. 22076381 10.1126/science.1203161

[42] Zhang P, Jamison K, Engel S, He B, He S (2011) Binocular rivalry requires visual attention. Neuron 71: 362–369. 21791293 10.1016/j.neuron.2011.05.035 PMC3175243

[43] Brascamp JW, Blake R (2012) Inattention abolishes binocular rivalry: perceptual evidence. Psychological Science 23: 1159–1167. 22933458 10.1177/0956797612440100

[44] Lamme VA, Spekreijse H (2000) Modulations of primary visual cortex activity representing attentive and conscious scene perception. Front Biosci 5: D232–243. 10704153 10.2741/lamme

[45] Lamme VAF, Zipser K, Spekreijse H (1998) Figure-ground activity in primary visual cortex is suppressed by anesthesia. Proceedings of the National Academy of Sciences of the United States of America 95: 3263–3268. 9501251 10.1073/pnas.95.6.3263 PMC19730

[46] Logothetis NK, Guggenberger H, Peled S, Pauls J (1999) Functional imaging of the monkey brain. Nat Neurosci 2: 555–562. 10448221 10.1038/9210

[47] Leopold DA, Murayama Y, Logothetis NK (2003) Very slow activity fluctuations in monkey visual cortex: Implications for functional brain imaging. Cerebral Cortex 13: 422–433. 12631571 10.1093/cercor/13.4.422

[48] Eckhorn R, Thomas U (1993) A new method for the insertion of multiple microprobes into neural and muscular tissue, including fiber electrodes, fine wires, needles and microsensors. J Neurosci Methods 49: 175–179. 8271837 10.1016/0165-0270(93)90121-7

[49] Tolias AS, Ecker AS, Siapas AG, Hoenselaar A, Keliris GA, et al (2007) Recording chronically from the same neurons in awake, behaving primates. Journal of Neurophysiology 98: 3780–3790. 17942615 10.1152/jn.00260.2007

[50] Robinson DA (1963) A Method of Measuring Eye Movement Using a Scleral Search Coil in a Magnetic Field. IEEE Trans Biomed Eng 10: 137–145. 14121113 10.1109/tbmel.1963.4322822

[51] Judge SJ, Richmond BJ, Chu FC (1980) Implantation of magnetic search coils for measurement of eye position: an improved method. Vision Res 20: 535–538. 6776685 10.1016/0042-6989(80)90128-5

[52] Crick F, Koch C (2003) A framework for consciousness. Nat Neurosci 6: 119–126. 12555104 10.1038/nn0203-119

[53] Dayan P (1998) A hierarchical model of binocular rivalry. Neural Comput 10: 1119–1135. 9654769 10.1162/089976698300017377

[54] Brascamp JW, Knapen TH, Kanai R, van Ee R, van den Berg AV (2007) Flash suppression and flash facilitation in binocular rivalry. J Vis 7: 12 11–12. 10.1167/7.12.12 17997654

[55] Silver MA, Logothetis NK (2007) Temporal frequency and contrast tagging bias the type of competition in interocular switch rivalry. Vision Res 47: 532–543. 17182075 10.1016/j.visres.2006.10.011

[56] Bhardwaj R, O'Shea RP, Alais D, Parker A (2008) Probing visual consciousness: rivalry between eyes and images. J Vis 8: 2 1–13. 10.1167/8.11.2 18831596

[57] Douglas RJ, Martin KAC (1991) A Functional Microcircuit for Cat Visual-Cortex. Journal of Physiology-London 440: 735–769. 10.1113/jphysiol.1991.sp018733 PMC1180177 1666655

[58] Mehta AD, Ulbert I, Schroeder CE (2000) Intermodal selective attention in monkeys. II: Physiological mechanisms of modulation. Cerebral Cortex 10: 359–370. 10769248 10.1093/cercor/10.4.359

[59] Mehta SD, Ulbert I, Schroeder CE (2000) Intermodal selective attention in monkeys. I: Distribution and timing of effects across visual areas. Cerebral Cortex 10: 343–358. 10769247 10.1093/cercor/10.4.343

[60] Buracas GT, Boynton GM (2007) The effect of spatial attention on contrast response functions in human visual cortex. Journal of Neuroscience 27: 93–97. 17202476 10.1523/JNEUROSCI.3162-06.2007 PMC6672290

[61] Corbetta M, Miezin FM, Dobmeyer S, Shulman GL, Petersen SE (1990) Attentional Modulation of Neural Processing of Shape, Color, and Velocity in Humans. Science 248: 1556–1559. 2360050 10.1126/science.2360050

[62] Huk AC, Ress D, Heeger DJ (2001) Neuronal basis of the motion aftereffect reconsidered. Neuron 32: 161–172. 11604147 10.1016/s0896-6273(01)00452-4

[63] Koch C, Tsuchiya N (2012) Attention and consciousness: related yet different. Trends in Cognitive Sciences 16: 103–105. 22154091 10.1016/j.tics.2011.11.012

[64] Guo K, Benson PJ, Blakemore C (2004) Pattern motion is present in V1 of awake but not anaesthetized monkeys. European Journal of Neuroscience 19: 1055–1066. 15009153 10.1111/j.1460-9568.2004.03212.x

[65] Lee SH, Blake R, Heeger DJ (2007) Hierarchy of cortical responses underlying binocular rivalry. Nature Neuroscience 10: 1048–1054. 17632508 10.1038/nn1939 PMC2615054

[66] Wilke M, Logothetis NK, Leopold DA (2003) Generalized flash suppression of salient visual targets. Neuron 39: 1043–1052. 12971902 10.1016/j.neuron.2003.08.003

[67] Lamme VAF, Zipser K, Spekreijse H (1998) Figure-ground activity in primary visual cortex is suppressed by anesthesia. Proc Natl Acad Sci U S A 95: 3263–3268. 9501251 10.1073/pnas.95.6.3263 PMC19730

[68] Lamme VAF, Spekreijse H (2000) Modulations of primary visual cortex activity representing attentive and conscious scene perception. Frontiers in Bioscience 5: D232–D243. 10704153 10.2741/lamme

[69] Alkire MT, Hudetz AG, Tononi G (2008) Consciousness and anesthesia. Science 322: 876–880. 18988836 10.1126/science.1149213 PMC2743249

[70] Super H, Spekreijse H, Lamme VAF (2001) Two distinct modes of sensory processing observed in monkey primary visual cortex (V1). Nat Neurosci 4: 304–310. 11224548 10.1038/85170

[71] Cohen MR, Maunsell JHR (2009) Attention improves performance primarily by reducing interneuronal correlations. Nat Neurosci 12: 1594-U1148. 19915566 10.1038/nn.2439 PMC2820564

[72] Mitchell JF, Sundberg KA, Reynolds JH (2009) Spatial Attention Decorrelates Intrinsic Activity Fluctuations in Macaque Area V4. Neuron 63: 879–888. 19778515 10.1016/j.neuron.2009.09.013 PMC2765230

[73] Ecker AS, Berens P, Keliris GA, Bethge M, Logothetis NK, et al (2010) Decorrelated Neuronal Firing in Cortical Microcircuits. Science 327: 584–587. 20110506 10.1126/science.1179867

[74] Ramalingam N, McManus JNJ, Li W, Gilbert CD (2013) Top-Down Modulation of Lateral Interactions in Visual Cortex. Journal of Neuroscience 33: 1773–1789. 23365217 10.1523/JNEUROSCI.3825-12.2013 PMC3711382

[75] Wilke M, Mueller KM, Leopold DA (2009) Neural activity in the visual thalamus reflects perceptual suppression. Proc Natl Acad Sci U S A 106: 9465–9470. 19458249 10.1073/pnas.0900714106 PMC2684842

[76] Goense JBM, Logothetis NK (2008) Neurophysiology of the BOLD fMRI signal in awake monkeys. Current Biology 18: 631–640. 18439825 10.1016/j.cub.2008.03.054

